# Age, gender, and racial differences in incidence and survival in primary CNS lymphoma

**DOI:** 10.1038/bjc.2011.357

**Published:** 2011-09-13

**Authors:** J L Villano, M Koshy, H Shaikh, T A Dolecek, B J McCarthy

**Affiliations:** 1 Department of Medicine, Section of Hematology/Oncology, University of Illinois at Chicago, 840 S. Wood Street, M/C 713, 820-E, room 839, Chicago, IL, USA; 2 Department of Radiation Therapy, University of Illinois at Chicago, Chicago, IL, USA; 3 Department of Radiation and Cellular Oncology, University of Chicago, Chicago, IL, USA; 4 Department of Epidemiology/Biostatistics, University of Illinois at Chicago, Chicago, IL, USA

**Keywords:** Lymphoma, brain tumour, epidemiology, incidence, trends

## Abstract

**Background:**

Primary central nervous system lymphoma (PCNSL) is a rare subtype of extranodal non-Hodgkin lymphoma that accounts for ∼4% of newly diagnosed central nervous system (CNS) tumours. The objective of this study was to analyse the epidemiology, incidence, and outcome of these rare tumours.

**Methods:**

Primary brain and CNS lymphoma cases were identified from the Surveillance, Epidemiology, and End Results (SEER) research data sets for the years 1980–2008 for analysis of trends in incidence and survival. SEER^*^Stat v. 7.0.4 software was used to analyse the data.

**Results:**

The overall incidence rate of PCNSL was 0.47 per 100 000 person-years. The incidence was significantly higher in males compared with females, blacks aged 0–49 years at diagnosis compared with whites, and whites aged 50 years and older at diagnosis compared with blacks. After a significant decline in incidence between 1995 and 1999, incidence rates rose slightly; those aged 75+ years at diagnosis had the most dramatic increase in incidence rates over time. Five-year survival rates were significantly higher in whites compared with blacks aged 0–49 years at diagnosis, but was primarily driven by white women aged 0–49 years.

**Conclusion:**

There is an increase in incidence of PCNSL in the elderly, and elderly blacks have lower incidence compared with white population. Survival remains poor and is negatively dominated by factors associated with HIV infection and advanced age.

Primary central nervous system lymphoma (PCNSL) is a rare disease that accounts for approximately 3–4% of newly diagnosed central nervous system (CNS) tumours (Hoffman *et al*, 2006). PCNSL is an uncommon subtype of extranodal non-Hodgkin lymphoma that involves the brain, leptomeninges, eyes, or spinal cord without evidence of systemic disease. The majority of PCNSL tumours are of the highly aggressive diffuse large cell subtypes, usually of B-cell phenotypic origin (Miller *et al*, 1994; Krogh-Jensen *et al*, 1995; Montesinos-Rongen *et al*, 2008). The World Health Organization classification of lymphoid and haematopoietic tissue tumours does not recognise PCNSL as a separate entity, as they are indistinguishable morphologically from systemic diffuse large B-cell lymphoma (DLBCL) (Campo *et al*, 2008).

The age-adjusted incidence rate has previously been reported at four cases per million persons per year, or ∼1200 cases per year in the United States (Hoffman *et al*, 2006). A prominent risk factor for the development of PCNSL is immunodeficiency, which includes congenital disorders, iatrogenic immunosuppression, and most notably, HIV. PCNSL is one of the four AIDS-defining malignancies and HIV infection carries a 3600-fold increased risk of developing the disease compared with the general population (Cote *et al*, 1996). There has been a significant increase in the incidence of PCNSL from the 1960s, which peaked in the mid-1990s (Schabet, 1999; Olson *et al*, 2002; Haldorsen *et al*, 2007). This is generally attributed to the increased incidence of HIV/AIDS and the subsequent development of effective anti-retroviral therapies. There also seems to be an increase in incidence of non-HIV-related PCNSL, which is associated with advanced age (Olson *et al*, 2002; Norden *et al*, 2011). Accurate differentiation, however, on a population basis from HIV/AIDS cases can be difficult, especially data sets early in the HIV infection history (1980–90s). Improved imaging and neurosurgical diagnostic services are also likely contributors to increased incidence (Abrey, 2009).

The outcome of PCNSL patients is dismal compared with patients with systemic DLBCL, where greater than 50% are long-term survivors. It is unclear if this is attributable to an intrinsic aggressive biological behaviour, the relatively immune-privileged CNS location or to some other yet undetermined cause. Outcomes are also different in HIV/AIDS-related PCNSL having a median survival of 2 months *vs* a year for non-HIV-related PCNSL (Norden *et al*, 2011).

We used the Surveillance, Epidemiology, and End Results (SEER) Programme of the National Cancer Institute to analyse the epidemiology and outcome of PCNSL, regardless of HIV status. Our goal was to perform a comprehensive analysis on this rare but very deadly brain cancer and relate our findings with systemic lymphomas, which is increasing in incidence in the elderly. We demonstrate that similar to systemic lymphomas there is a clear increase in incidence of PCNSL in the elderly and this increase is enlarging over time.

## Materials and Methods

The SEER programme has collected population-based incidence and survival data on all primary malignant cancers since 1973. For both the descriptive incidence data and the survival analyses, the SEER April 2011 research data for 17 registries were used to analyse data for all primary brain and CNS lymphomas diagnosed between 2000 and 2008 (SEER, 2011b, 2011c). The SEER April 2011 research data for nine registries were used to analyse trends in incidence data for all primary brain and CNS lymphomas diagnosed between 1980 and 2008 (SEER, 2011a).

The set of ‘all primary malignant brain tumours’ was defined by using the International Classification of Diseases for Oncology (ICD-O-3) site codes of C70.0—C72.9 and C75.1—C75.3, and a behaviour code of 3 (i.e., malignant; Fritz, 2000). Lymphomas were selected using ICD-O-3 histology codes 9590–9599 and 9670–9729.

Incidence rates and frequencies were estimated using SEER^*^Stat v. 7.0.4 (Surveillance Research Program, 2010). Incidence rates were generated for all brain lymphomas overall and by age groups, race, gender, and by diagnosis year to show trends over time. Rates were per 100 000 person-years (p-y) and were age-adjusted to the year 2000 US standard population. Relative survival rates were estimated from the SEER 17-registries research data set using SEER^*^Stat v. 7.0.4. Relative survival was defined as the observed probability of survival adjusted for the expected survival rate of the US population for that age, sex, and race. Kaplan–Meier relative survival rates were calculated by gender and race group. Survival time was calculated from the date of diagnosis to the date of death or last contact. Patients who were alive were censored at the date of last contact. A Z-score was calculated to compare two survival curves using SEER^*^Stat v. 7.0.4 and from which a *P*-value was then estimated (Brown, 1983).

Effects of age, sex, and race on PCNSL survival time within two selected age groups (0–49 years and 50+ years) were analysed using proportional hazards regression models (Hosmer *et al*, 2008). The SEER^*^Stat Joinpoint Regression Program v. 3.4.3 was used to identify changes in the slope of the lines in incidence rate trends over time using permutation rates in a log linear model (Kim *et al*, 2000; Montesinos-Rongen *et al*, 2008). A maximum of three joinpoints were allowed for the number of years included in the analysis.

## Results

The SEER 17 registries research data set comprised 3100 cases of PCNSL diagnosed between 2000 and 2008 (Table 1). The most frequently reported primary sites were the brain lobes (33%) and brain, NOS (not otherwise specified; 34% data not shown). The overall incidence of CNS PCNSL was 0.47 per 100 000 p-y (Table 1), with a significantly higher incidence in males (0.55 per 100 000 p-y) compared with females (0.39 per 100 000 p-y). Incidence rates increased with increasing age at diagnosis (Table 1), and demonstrated a similar pattern in both males and females although males had significantly higher incidence rates of PCNSL in the 0–34, 35–44, 45–54, 55–64, and 75+ age at diagnosis year groups than females (data not shown). Overall, incidence rates were similar between racial groups (Table 1). However, by race and age group, the incidence in whites <50 years at diagnosis was significantly lower than in blacks <50 years. Conversely, whites ⩾50 years had a significantly higher incidence of CNS PCNSL than blacks ⩾50 years. This pattern was observed for both males and females, with both black males and females having higher incidence rates in those diagnosed before age 50 years, but lower incidence rates after age 50 years than white males and females (Table 1).

**Figure 1:**
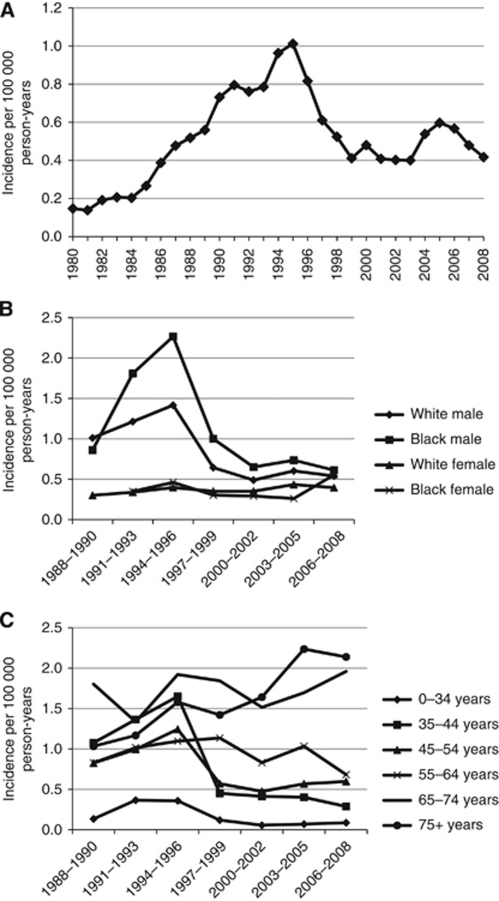
Incidence rate trends over time for (**A**) CNS lymphomas; SEER nine registries research data, 1980–2008; (**B**) CNS lymphomas by gender and race; SEER, 1988–2008; and (**C**) CNS lymphomas by age group at diagnosis; SEER, 1988–2008.

The average annual age-adjusted incidence rate of PCNSLs increased significantly over time through the mid-1990s (likely due in part to onset of the AIDS epidemic), but has since decreased (Figure 1A). Using joinpoint, three changes in the slope of the incidence line were identified. First, the incidence rate for the years 1980 to 1990 significantly increased with an annual percent change (APC) of 18.7. Then, a non-significant reduction in the slope of the line was observed as the increase peaked in 1995 (APC=6.9). Between 1995 and 1999, the incidence rate of CNS PCNSL significantly decreased (APC=−19.0), but since then, a slight non-significant increase is suggested (APC=2.1). Incidence rates in both black and white males increased during the mid-1990s, but have since decreased, whereas the incidence rates in white females have remained comparatively stable over time (Figure 1B). Sample sizes were too small to identify patterns in race, gender, and age subgroups by individual years of diagnosis. Incidence rates for the four youngest age groups increased through the mid-1990s, but have since decreased (Figure 1C). Conversely, in the 65- to 74-year-age group, incidence rates for PCNSL have increased since diagnosis year 2000. The most significant increase in the incidence rates for PCNSL over time has occurred in the oldest adults (aged 75+ years), although the incidence may be starting to decease in the most recent time period (Figure 1C).

**Figure 2:**
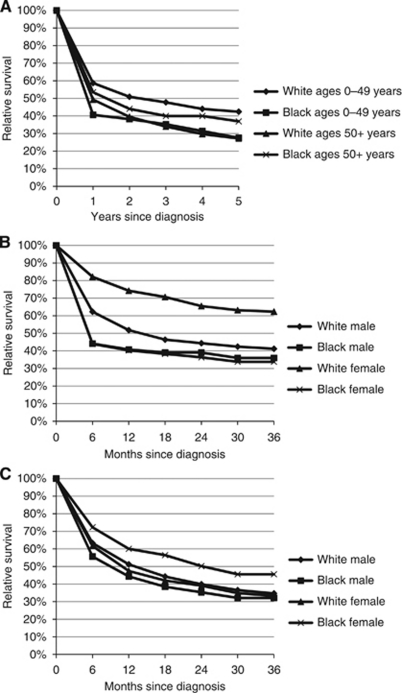
Relative survival for CNS lymphomas by (**A**) age group (0–49 years, 50+ years) and race, (**B**) by gender and race for cases diagnosed before 50 years of age, and (**C**) by gender and race for cases diagnosed at ages 50 or older years; SEER, 2000–2008.

For subjects diagnosed between 2000 and 2008, overall 1-, 2-, and 5-year relative survival estimates were 51.4%, 42.6%, and 31.2%, respectively, with a median relative survival of 14 months (*n*=2614; data not shown). By race and age group, whites aged 0–49 years at diagnosis had significantly better survival than blacks aged 0–49 years (5-year relative survival=42.4% *vs* 27.5%, respectively; *P*-value at 5 years <0.0001; Figure 2A). For those aged 50 years or older at diagnosis, the relative survival was lower in whites compared with blacks (5-year relative survival=27.2% *vs* 36.9%, respectively), although this difference was not statistically significant (*P*-value=0.20; Figure 2A). For those aged 0–49 years at diagnosis, white females (*n*=163) had significantly (*P*<0.0001) better relative survival at 36 months than all other race and gender categories, whereas white (*n*=372) males had better relative survival at 36 months than black males (*P*=0.03) and black females (*P*=0.06). Relative survival at 36 months did not differ between black males (*n*=118) and black females (*n*=59) (Figure 2B). Conversely, for those aged 50 years or older at diagnosis, black females (*n*=50) had somewhat better survival at 36 months (*P*=0.08) than white females (*n*=802), although the sample size was too small to draw any definitive conclusions. Survival between all other race and gender categories did not differ (white male *n*=763 and black male *n*=36; Figure 2C).

Table 2 displays results from the proportional hazards regression analysis. In those with PCNSL aged 0–49 years, age, sex, and race were all significantly associated with survival. That is, advancing age, male gender, and black race were associated with decreased survival time. Conversely, in those aged 50+ years, only increasing age was associated with lower survival time.

## Discussion

Depending on the period of analysis during the HIV/AIDS epidemic and therapeutic history investigators have described both an increase and decrease in incidence of PCNSL (Fine and Mayer, 1993; Kadan-Lottick *et al*, 2002; Olson *et al*, 2002; Hoffman *et al*, 2006). They have also differentiated HIV/AIDS-related from non-AIDS-related cases, using slightly different methodologies (Olson *et al*, 2002; Panageas *et al*, 2005; Norden *et al*, 2011). Our result demonstrate that the incidence of PCNSL have recently been stable, and is similar to the CBTRUS annual report, which includes data from 48 population-based cancer registries in the United States (including the SEER registries included in this manuscript) for diagnosis years 2004–2007 (CBTRUS, 2011). The overall incidence is 0.47 per 100 000 p-y, with higher rates in males, but similar overall incidence rates by race. However, when incidence rates were estimated by both race and age at diagnosis, there are notable differences.

When evaluating by age groups, there is an increase in incidence with advancing age, with the 75+ group having the highest rate, and in recent years the difference has widened. This is consistent with an increase in incidence of advanced age (presumed non-HIV)-related PCNSL that have been reported (Olson *et al*, 2002; Norden *et al*, 2011). Other systemic lymphomas have also increased in incidence in the elderly. Data from SEER demonstrates that the rate of DLBCL in the elderly increased 1.4% per year from 1992 to 2001 (Morton *et al*, 2006). The increased incidence of DLBCL in the elderly has been attributed to a possible reduction in immunological surveillance or an increased number of somatic mutations that accrue over a lifetime. It is possible that the increasing incidence of PCNSL in the elderly could be attributed to similar mechanisms (Westin and Longo, 2004; Muller *et al*, 2005). It is also possible that this increase is reflecting a growing elderly population receiving more immunosuppressive therapies, for example, for organ transplantation and autoimmune diseases.

HIV/AIDS disproportionately affects blacks, by greater than an order of magnitude than other race groups, as well as young adults (Morris *et al*, 2006). Black males in the mid 1990s had nearly twice the incidence of white males, but the most recent years of analysis, 2006–2008, no differences are present. This is consistent with results that the cancer burden of HIV infection in a 15-year period, 1991–2005, has changed markedly, with there being three-fold less AIDS-defining cancers, which includes PCNSL, with a concomitant three-fold increase in non-AIDS-defining cancers (Shiels *et al*, 2011). This is attributed to an aging population, which increases the risk for cancer, as well as having improved therapies that have increased the HIV population in the US four-fold during this 15-year period.

Analysis by a broad age group of 0–49 and 50+ years demonstrate differences in incidence by race. The 0–49-year group of black males had greater than twice the incidence of white males, whereas the 50+-year group of white males had greater than twice the incidence of black males. Although the number of cases was less, the same pattern was present for black females. This indicates that blacks have a lower incidence of the advanced age (presumed non-HIV)-related PCNSL, which is consistent with the lower incidence of systemic lymphomas in blacks (Ghafoor *et al*, 2002).

Consistent with other series, our analysis of overall survival reveals that PCNSL is associated with poor long-term outcomes (Fine and Mayer, 1993; Pulido *et al*, 2009; Norden *et al*, 2011). However, as we restricted our analyses to the most recent time period, our survival rates are higher than previous studies and likely reflect improvements in treatment (O’Brien and Seymour, 2009; Pulido *et al*, 2009; Chamberlain and Johnston, 2010; Thiel *et al*, 2010; Norden *et al*, 2011). Independent predictors of mortality have been known to be the male sex, being HIV positive, black, and advanced age over 60 years (Pulido *et al*, 2009; Norden *et al*, 2011; Thiel *et al*, 2011). The contribution of HIV/AIDS is significant as median overall survival is only 2 months *vs* 12 months in the non-HIV patient (Norden *et al*, 2011). Our analysis also demonstrates the significant role of HIV infection has on outcomes as the 0–49 group, where HIV is more prevalent, have advanced age, male sex, and being black associated with decreased survival, but in the 50+ group only increasing age is associated with decreased survival.

There are limitations to our analysis. The SEER data set may not reflect true incidence due to ascertainment bias from improved diagnostic techniques including increased utilisation of stereotactic biopsies, flow-cytometry-based immunochemistry and PCR techniques on tissue biopsies or cells from the cerebrospinal fluid (Abrey, 2009). This could explain an increase in incidence in the elderly, where previously a diagnostic procedure may not have been performed. It is also possible that our findings of an increase in incidence in the elderly is reflecting a greater use of immunosuppressive therapies or a subset of longer surviving patients with lower CD4 counts, who are more susceptible to the development of PCNSL. There are other limitations as our database, SEER, lacks coding for risk factors associated with PCNSL, including congenital disorders, iatrogenic immunosuppression, and HIV status. Furthermore, we have no information on performance status and limited information on mode of therapy including no information about use of chemotherapy.

In summary, there is an increase in incidence of PCNSL in the elderly, and elderly blacks have lower incidence, which is in keeping with their lower incidence of systemic lymphomas. Survival remains poor and is negatively dominated by factors associated with HIV infection and advanced age. These findings demonstrate the benefit of analysing population-based registries to contribute relevant clinical information on rare brain tumours.

## Figures and Tables

**Table 1:** Incidence rates (per 100 000) for primary CNS lymphomas by gender, race, and age group; SEER 17 registries research data, 2000–2008

	***N***	**Incidence rate (95% CI)**
All CNS lymphomas	3100	0.47 (0.45–0.49)
		
*Gender*		
Male	1697	0.55 (0.53–0.58)
Female	1403	0.39 (0.37–0.42)
		
*Race group*		
White	2523	0.47 (0.45–0.49)
Black	287	0.41 (0.36–0.46)
Other/unknown	290	0.46 (0.41–0.52)
		
*Age group (years)*		
0–34	260	0.08 (0.07–0.09)
35–44	343	0.33 (0.29–0.36)
45–54	487	0.51 (0.46–0.56)
55–64	581	0.91 (0.84–0.99)
65–74	712	1.78 (1.65–1.92)
75+	717	1.90 (1.77–2.05)
		
*Age group by race and gender*		
0–49 years	842	0.17 (0.16–0.18)
White	591	0.16 (0.14–0.17)
Male	417	0.22 (0.20–0.24)
Female	174	0.09 (0.08–0.11)
Black	189	0.34 (0.29–0.39)
Male	126	0.47 (0.39–0.56)
Female	63	0.21 (0.16–0.27)
50+ years	2258	1.25 (1.20–1.31)
White	1932	1.30 (1.24–1.36)
Male	949	1.43 (1.34–1.52)
Female	983	1.20 (1.13–1.28)
Black	98	0.61 (0.49–0.75)
Male	42	0.57 (0.41–0.79)
Female	56	0.62 (0.47–0.81)

Abbreviations: CI=confidence interval; CNS=central nervous system; SEER=Surveillance Epidemiology and End Results.

**Table 2:** Estimated hazard ratios for primary CNS lymphoma within selected age groups for age, sex, and race using proportional hazards regression

	**Hazard ratios**	**95.0% CI**	***P*-value**
*Ages 0–49 years*			
Age (continuous)	1.02	(1.01–1.03)	<0.0001
Sex (male/female)	0.71	(0.57–0.88)	0.002
Race (white/black)	1.63	(1.32–2.02)	<0.0001
			
*Ages 50+ years*			
Age (continuous)	1.04	(1.04–1.05)	<0.0001
Sex (male/female)	0.95	(0.84–1.06)	0.340
Race (white/black)	1.01	(0.77–1.34)	0.922

Abbreviations: CI=confidence interval; CNS=central nervous system.

## References

[bib1] Abrey LE (2009) Primary central nervous system lymphoma. Curr Opin Neurol 22: 675–680 19741527 10.1097/WCO.0b013e328332533b

[bib2] Brown CC (1983) The statistical comparison of relative survival rates. Biometrics 39: 941–948 6671129

[bib3] Campo E, Swerdlow SH, Harris NL, Pileri S, Stein H, Jaffe ES (2008) The 2008 WHO classification of lymphoid neoplasms and beyond: evolving concepts and practical applications. Blood 117(19): 5019–5032 10.1182/blood-2011-01-293050 PMC3109529 21300984

[bib4] CBTRUS (2011) CBTRUS Statistical Report: Primary Brain and Central Nervous System Tumors Diagnosed in the United States in 2004–2007. Central Brain Tumor Registry of the United States

[bib5] Chamberlain MC, Johnston SK (2010) High-dose methotrexate and rituximab with deferred radiotherapy for newly diagnosed primary B-cell CNS lymphoma. Neuro Oncol 12: 736–744 20511181 10.1093/neuonc/noq011 PMC2940660

[bib6] Cote TR, Manns A, Hardy CR, Yellin FJ, Hartge P (1996) Epidemiology of brain lymphoma among people with or without acquired immunodeficiency syndrome. AIDS/Cancer Study Group. J Natl Cancer Inst 88: 675–679 8627644 10.1093/jnci/88.10.675

[bib7] Fine HA, Mayer RJ (1993) Primary central nervous system lymphoma. Ann Intern Med 119: 1093–1104 8239229 10.7326/0003-4819-119-11-199312010-00007

[bib8] Fritz AG, World Health Organization (2000) International Classification of Diseases for Oncology : ICD-O, 3rd edn World Health Organization: Geneva

[bib9] Ghafoor A, Jemal A, Cokkinides V, Cardinez C, Murray T, Samuels A, Thun MJ (2002) Cancer statistics for African Americans. CA Cancer J Clin 52: 326–341 12469762 10.3322/canjclin.52.6.326

[bib10] Haldorsen IS, Krossnes BK, Aarseth JH, Scheie D, Johannesen TB, Mella O, Espeland A (2007) Increasing incidence and continued dismal outcome of primary central nervous system lymphoma in Norway 1989–2003: time trends in a 15-year national survey. Cancer 110: 1803–1814 17721992 10.1002/cncr.22989

[bib11] Hoffman S, Propp JM, McCarthy BJ (2006) Temporal trends in incidence of primary brain tumors in the United States, 1985–1999. Neuro Oncol 8: 27–37 16443945 10.1215/S1522851705000323 PMC1871920

[bib12] Hosmer DW, Lemeshow S, May S (2008) Applied Survival Analysis: Regression Modeling of Time-to-Event Data, 2nd edn Wiley-Interscience: Hoboken, NJ

[bib13] Kadan-Lottick NS, Skluzacek MC, Gurney JG (2002) Decreasing incidence rates of primary central nervous system lymphoma. Cancer 95: 193–202 12115333 10.1002/cncr.10643

[bib14] Kim HJ, Fay MP, Feuer EJ, Midthune DN (2000) Permutation tests for joinpoint regression with applications to cancer rates. Stat Med 19: 335–351 10649300 10.1002/(sici)1097-0258(20000215)19:3<335::aid-sim336>3.0.co;2-z

[bib15] Krogh-Jensen M, D′Amore F, Jensen MK, Christensen BE, Thorling K, Pedersen M, Johansen P, Boesen AM, Andersen E (1995) Clinicopathological features, survival and prognostic factors of primary central nervous system lymphomas: trends in incidence of primary central nervous system lymphomas and primary malignant brain tumors in a well-defined geographical area. Population-based data from the Danish Lymphoma Registry, LYFO, and the Danish Cancer Registry. Leuk Lymphoma 19: 223–233 8535213 10.3109/10428199509107892

[bib16] Miller DC, Hochberg FH, Harris NL, Gruber ML, Louis DN, Cohen H (1994) Pathology with clinical correlations of primary central nervous system non-Hodgkin's lymphoma. The Massachusetts General Hospital experience 1958–1989. Cancer 74: 1383–1397 8055462 10.1002/1097-0142(19940815)74:4<1383::aid-cncr2820740432>3.0.co;2-1

[bib17] Montesinos-Rongen M, Brunn A, Bentink S, Basso K, Lim WK, Klapper W, Schaller C, Reifenberger G, Rubenstein J, Wiestler OD, Spang R, Dalla-Favera R, Siebert R, Deckert M (2008) Gene expression profiling suggests primary central nervous system lymphomas to be derived from a late germinal center B cell. Leukemia 22: 400–405 17989719 10.1038/sj.leu.2405019 PMC6053313

[bib18] Morris M, Handcock MS, Miller WC, Ford CA, Schmitz JL, Hobbs MM, Cohen MS, Harris KM, Udry JR (2006) Prevalence of HIV infection among young adults in the United States: results from the Add Health study. Am J Public Health 96: 1091–1097 16670236 10.2105/AJPH.2004.054759 PMC1470623

[bib19] Morton LM, Wang SS, Devesa SS, Hartge P, Weisenburger DD, Linet MS (2006) Lymphoma incidence patterns by WHO subtype in the United States, 1992–2001. Blood 107: 265–276 16150940 10.1182/blood-2005-06-2508 PMC1895348

[bib20] Muller AM, Ihorst G, Mertelsmann R, Engelhardt M (2005) Epidemiology of non-Hodgkin's lymphoma (NHL): trends, geographic distribution, and etiology. Ann Hematol 84: 1–12 15480663 10.1007/s00277-004-0939-7

[bib21] Norden AD, Drappatz J, Wen PY, Claus EB (2011) Survival among patients with primary central nervous system lymphoma, 1973–2004. J Neurooncol 101: 487–493 20556477 10.1007/s11060-010-0269-7

[bib22] O’Brien PC, Seymour JF (2009) Progress in primary CNS lymphoma. Lancet 374: 1477–1478 19767091 10.1016/S0140-6736(09)61488-4

[bib23] Olson JE, Janney CA, Rao RD, Cerhan JR, Kurtin PJ, Schiff D, Kaplan RS, O′Neill BP (2002) The continuing increase in the incidence of primary central nervous system non-Hodgkin lymphoma: a surveillance, epidemiology, and end results analysis. Cancer 95: 1504–1510 12237919 10.1002/cncr.10851

[bib24] Panageas KS, Elkin EB, DeAngelis LM, Ben-Porat L, Abrey LE (2005) Trends in survival from primary central nervous system lymphoma, 1975–1999: a population-based analysis. Cancer 104: 2466–2472 16240449 10.1002/cncr.21481

[bib25] Pulido JS, Vierkant RA, Olson JE, Abrey L, Schiff D, O’Neill BP (2009) Racial differences in primary central nervous system lymphoma incidence and survival rates. Neuro Oncol 11: 318–322 19273630 10.1215/15228517-2008-103 PMC2718976

[bib26] Schabet M (1999) Epidemiology of primary CNS lymphoma. J Neurooncol 43: 199–201 10563423 10.1023/a:1006290032052

[bib27] Shiels MS, Pfeiffer RM, Gail MH, Hall HI, Li J, Chaturvedi AK, Bhatia K, Uldrick TS, Yarchoan R, Goedert JJ, Engels EA (2011) Cancer burden in the HIV-infected population in the United States. J Natl Cancer Inst 103: 753–762 21483021 10.1093/jnci/djr076 PMC3086877

[bib28] SEER (2011a) Surveillance, Epidemiology, and End Results (SEER) Program (http://www.seer.cancer.gov) SEER*Stat Database: Incidence - SEER 9 Regs Research Data, Nov 2010 Sub (1973–2008) <Katrina/Rita Population Adjustment> - Linked To County Attributes - Total US, 1969–2009 Counties, National Cancer Institute, DCCPS, Surveillance Research Program, Cancer Statistics Branch, released April 2011, based on the November 2010 submission

[bib29] SEER (2011b) Surveillance, Epidemiology, and End Results (SEER) Program (http://www.seer.cancer.gov) SEER*Stat Database: Incidence - SEER 17 Regs Research Data + Hurricane Katrina Impacted Louisiana Cases, Nov 2010 Sub (2000–2008) <Katrina/Rita Population Adjustment> - Linked To County Attributes - Total US, 1969-2009 Counties, National Cancer Institute, DCCPS, Surveillance Research Program, Cancer Statistics Branch, released April 2011, based on the November 2010 submission

[bib30] SEER (2011c) Surveillance, Epidemiology, and End Results (SEER) Program (http://www.seer.cancer.gov) SEER*Stat Database: Incidence - SEER 17 Regs Research Data + Hurricane Katrina Impacted Louisiana Cases, Nov 2010 Sub (1973–2008 varying) - Linked To County Attributes - Total US, 1969–2009 Counties, National Cancer Institute, DCCPS, Surveillance Research Program, Cancer Statistics Branch, released April 2011, based on the November 2010 submission

[bib31] Surveillance Research Program (2010) National Cancer Institute SEER^*^Stat software (http://www.seer.cancer.gov/seerstat) version 7.0.4

[bib32] Thiel E, Korfel A, Martus P, Kanz L, Griesinger F, Rauch M, Roth A, Hertenstein B, von Toll T, Hundsberger T, Mergenthaler HG, Leithauser M, Birnbaum T, Fischer L, Jahnke K, Herrlinger U, Plasswilm L, Nagele T, Pietsch T, Bamberg M, Weller M (2010) High-dose methotrexate with or without whole brain radiotherapy for primary CNS lymphoma (G-PCNSL-SG-1): a phase 3, randomised, non-inferiority trial. Lancet Oncol 11: 1036–1047 20970380 10.1016/S1470-2045(10)70229-1

[bib33] Westin EH, Longo DL (2004) Lymphoma and myeloma in older patients. Semin Oncol 31: 198–205 15112150 10.1053/j.seminoncol.2003.12.030

